# Does the PI3K pathway promote or antagonize regulatory T cell development and function?

**DOI:** 10.3389/fimmu.2012.00244

**Published:** 2012-08-14

**Authors:** Dalya R. Soond, Elizabeth C. M. Slack, Oliver A. Garden, Daniel T. Patton, Klaus Okkenhaug

**Affiliations:** ^1^Laboratory of Lymphocyte Signalling and Development, Babraham InstituteCambridge, UK; ^2^Regulatory T Cell Laboratory, Infection and Immunity Research Group, Department of Veterinary Clinical Sciences, The Royal Veterinary College, Camden CampusLondon, UK; ^3^Department of Microbiology and Immunology, Life Sciences Institute, The University of British ColumbiaVancouver, BC, Canada

**Keywords:** Akt, autoimmunity, Foxo, inflammation, mTOR, PI3K, T cell, Treg

## Abstract

Regulatory T cells (Tregs) prevent autoimmunity and inflammation by suppressing the activation of other T cells and antigen presenting cells. The role of phosphoinositide 3-kinase (PI3K) signaling in Treg is controversial. Some studies suggest that inhibition of the PI3K pathway is essential for the development of Tregs whereas other studies have shown reduced Treg numbers and function when PI3K activity is suppressed. Here we attempt to reconcile the different studies that have explored PI3K and the downstream effectors Akt, Foxo, and mTOR in regulatory T cell development and function and discuss the implications for health and therapeutic intervention.

## Introduction

The class I phosphoinositide 3-kinases (PI3Ks) consist of heterodimers between one regulatory and one catalytic subunit. The Class IA catalytic subunit isoforms (p110α, p110β, and p110δ) can be activated by tyrosine kinase-associated and sometimes G protein-coupled receptors while the Class IB isoform (p110γ) can only be activated by G protein-coupled receptors. Each class I PI3K isoform uses PtdIns(4,5)P_2_ as its preferred substrate to generate the second messenger PtdIns(3,4,5)P_3_, which helps activate PH domain-containing signaling proteins. Key downstream targets include Akt, PDK-1, and Tec family kinases (Okkenhaug and Fruman, 2010; So and Fruman, 2012).

PtdIns(3,4,5)P_3_ generation recruits and co-localizes Akt and PDK1 to the plasma membrane. PDK1 can then phosphorylate Akt on Thr^308^. A second phosphorylation at Ser^473^ is required for optimal Akt activity. This residue is phosphorylated by the rapamycin-insensitive mTOR/Rictor complex (mTORC2) and de-phosphorylated by PHLPP. Akt, together with numerous other upstream regulators, can then indirectly contribute to activation of the rapamycin-sensitive mTOR/Raptor complex (mTORC1). Akt also phosphorylates the transcription factor FOXO, which leads to its exclusion from the nucleus, thus altering T cell homeostasis and trafficking (Kerdiles et al., 2009; Finlay and Cantrell, 2010).

PtdIns(3,4,5)P_3_ signaling is terminated by two classes of phosphatases. Pten dephosphorylates PtdIns(3,4,5)P_3_ on the D3 position to maintain resting levels of PtdIns(4,5)P_2_. SHIP phosphatases dephosphorylate PtdIns(3,4,5)P_3_ on the D5 position to generate PtdIns(3,4)P_2_, which has signaling properties of its own (Okkenhaug and Fruman, 2010; So and Fruman, 2012).

The PI3K isoform p110δ was shown to be the dominant isoform downstream of the T cell receptor (TCR), the co-stimulatory receptor ICOS, and the IL-2 receptor (Okkenhaug et al., 2002, 2006; Rolf et al., 2010; Soond et al., 2010; Macintyre et al., 2011). Consequently, p110δ controlled proliferation, cytokine production, differentiation into helper T cells (Th) subsets, and trafficking (Okkenhaug et al., 2002, 2006; Nashed et al., 2007; Garcon et al., 2008; Jarmin et al., 2008; Sinclair et al., 2008; Liu et al., 2009; Rolf et al., 2010; Soond et al., 2010; Macintyre et al., 2011). The p110γ isoform of PI3K is required for migration toward inflammatory chemokines and memory T cell survival (Barber et al., 2006; Martin et al., 2008; Thomas et al., 2008). Conversely, T cells lacking Pten are hypersensitive to TCR and IL-2 signaling leading to augmented Th cell functions, autoimmunity, and leukemia (Suzuki et al., 2001; Buckler et al., 2006; Liu et al., 2010; Guo et al., 2011; Soond et al., 2012).

The role of the PI3K pathway in T cells has been addressed experimentally by either inhibiting PI3K signaling (e.g. by inactivating PI3K or downstream proteins such as PDK1 or mTOR) or by increasing PI3K signaling (e.g. by deleting Pten, SHIP or Foxo, or by overexpressing membrane-targeted Akt). Although there seems to be a clear positive role for the PI3K pathway in inducing the activation, differentiation, and maintenance of Th cells, data regarding its precise effect in regulatory T cells (Tregs) appears contradictory. In this review, we will briefly summarize what is known about the development and function of Tregs, describe how Tregs are regulated by the PI3K pathway, and propose how conflicting data can be reconciled.

## Tregs suppress immune responses

Tregs are defined as the 5–10% of CD4^+^ T cells that express the transcription factor Foxp3. Foxp3 expression is both necessary and sufficient to confer suppressive ability to Tregs. Tregs prevent autoimmunity, restrain the responses to infectious agents, aid maternal tolerance toward fetuses and block tumor immunity. Their importance has been shown in cases where Foxp3 is lost or attenuated such as IPEX syndrome in humans or *scurfy* mice, where by multi-organ autoimmunity and inflammation rapidly develops, leading to death of the organism (Bennett et al., 2001; Brunkow et al., 2001; Wildin et al., 2001; Yamaguchi et al., 2011; Josefowicz et al., 2012a). Depletion of Foxp3^+^ cells in adult mice also leads to fatal disease, highlighting their role in preventing responses throughout the life of the organism (Kim et al., 2007).

## Treg develop in the thymus and periphery

The majority of Treg cells are generated in the thymus and are termed “natural Tregs” (nTregs). Commitment to this lineage occurs in two steps. First, TCR signaling in CD4^+^CD8^+^ double positive T cells poises them to express Foxp3, which then occurs in a second IL-2-dependent but TCR-independent step (Lio and Hsieh, 2008). The amount of TCR signaling required for the positive selection of Tregs is higher than for conventional T cells, but less than is required for negative selection. Hence, the TCRs expressed by Tregs tend to have higher affinity for self-peptide/MHC complexes than those expressed by Th cells (Hsieh et al., 2012).

Tregs can also be generated outside the thymus from naïve CD4^+^ T cells. These “induced Tregs” (iTregs) develop when the TCR is activated under immunosuppressive conditions in the presence of TGFβ1 (Chen et al., 2003), indoleamine 2,3-dioxygenase, or other amino acid metabolizing enzymes (Chen et al., 2008; Chung et al., 2009; Cobbold et al., 2009), or when T cells are activated by Ag at low doses or low affinity antigen (Daniel et al., 2010; Gottschalk et al., 2010). Although there is a lack of reliable markers to unequivocally track the survival of iTreg, it is estimated that iTreg represent only a small proportion of the total Treg population under homeostatic conditions (Zheng et al., 2010; Josefowicz et al., 2012b).

The Foxp3 gene locus contains a promoter and three additional conserved non-coding DNA sequences (CNS1-3) which include binding sites for diverse transcription factors such as NFAT, NF-κB, AP1, STAT5, Cbf, Runx, Foxo, Foxp3, SMAD, and other factors (Merkenschlager and Von Boehmer, 2010; Zheng et al., 2010). The number of elements involved in *Foxp3* regulation suggests this locus is tightly controlled and highly responsive to context-dependent cues. Not all of these transcription factors or promoter regions are required for *Foxp3* transcription at all times. CNS3, which binds c-Rel but not other members of the NF-κB transcription factor family, is considered to be a pioneer element accessible in Treg precursors. Consistent with this, Treg development in the thymus is blocked in the absence of CNS3 or c-Rel (Isomura et al., 2009; Visekruna et al., 2010; Zheng et al., 2010). The CNS1 element binds Smad3 and—along with CNS3—is required for TGF-β-induced conversion to iTreg. CNS1 deficiency primarily affects Treg numbers at environmentally exposed tissues such as the intestine and lung where iTregs are most frequently found, but is dispensable for nTreg development (Zheng et al., 2010; Josefowicz et al., 2012b). CNS2, but not CNS3, is required for maintenance of Treg in the periphery (Zheng et al., 2010). This is of interest as CNS2 binds Foxp3 protein and may hence stabilize the lineage as part of a positive feedback loop. Multiple regulatory inputs mean that a genetic lesion may alter Treg numbers by affecting the development and/or maintenance of Foxp3.

Fate-mapping studies have shown somewhat conflicting results with regards to Treg plasticity. While some studies had suggested that some Treg can be re-differentiated to other Th lineages (Tsuji et al., 2009; Zhou et al., 2009), further studies suggest that the expression of Foxp3 is highly stable and irreversible (Rubtsov et al., 2010; Miyao et al., 2012). However, it is possible that a certain percentage of Th cells express Foxp3 transiently, but subsequently are diverted to other lineages (Komatsu et al., 2009). Consistent with this notion, when expression of Foxp3 was intentionally destabilized, the Foxp3^low^ T cells were subverted into Th2 cells that caused disease (Wan and Flavell, 2007). It should be noted however, that fully committed Foxp3^+^ Treg can co-express transcription factors associated with other T cells lineages, such as Tbet, Gata3, IRF4, or Bcl6. This may help adapt the Foxp3^+^ Treg to limit particular types of immune responses, for instance by targeting them to the correct anatomical location (Josefowicz et al., 2012a).

## PI3K activity supresses the development of nTreg

How does the PI3K pathway affect development of nTregs in the thymus? The p110δ^D910A^ mouse, in which p110δ is inactivated by point mutation, showed increased proportions of Tregs in the thymus (Patton et al., 2006). There were more immature as well as mature thymic Tregs, suggesting that the increased Foxp3^+^ population reflects enhanced development of Foxp3^+^ T cells rather than accumulation of mature Treg that fail to emigrate to the periphery (Patton et al., 2006). Consistent with a negative role for PI3K in nTreg development, retroviral expression of oncogenic Akt reduced the number of nTregs (Haxhinasto et al., 2008). Treg numbers were also dramatically decreased in the thymi of mice lacking Foxo1 and Foxo3 expression in T cells, although this defect resolved as the mice aged (Kerdiles et al., 2010; Ouyang et al., 2010). Foxo transcription factors have been found to directly bind CNS1 and CNS3 regions of the *Foxp3* locus, providing a direct mechanism for their role in nTreg and iTreg development (Harada et al., 2010; Ouyang et al., 2010). By contrast, in mice lacking mTOR in T cells there was no difference in nTreg (Delgoffe et al., 2009). The simplest conclusion from these experiments is that the PI3K p110δ antagonizes nTreg development by activating Akt, leading to the exclusion of Foxo from the nucleus.

In apparent contradiction to these results, mice lacking PDK1 in T cells had reduced Treg numbers in the thymus (Park et al., 2010). It is important to appreciate, however, that PDK1 regulates multiple protein kinase C isoforms independently of PI3K and Akt (Mcmanus et al., 2004; Waugh et al., 2009); hence the reduced numbers of thymic Treg in these mice might be a consequence of impaired c-Rel activation (which depends on PKC activity) rather than interrupted PI3K signaling.

## PI3K signals can enhance or block peripheral conversion of naïve CD4^+^ T cells

We found that TGF-β1-stimulated iTreg conversion was reduced when we used the pan-PI3K inhibitor PI-103, the p110δ-selective inhibitor IC87114 or rapamycin (Patton et al., 2011); however, others have observed enhanced iTreg conversion upon addition of PI3K or mTOR inhibitors (Harada et al., 2010; Patterson et al., 2011). We do not have an explanation for these differences other than it may depend on the amount of costimulation provided in the conversion cultures, as in some cases CD28 signals can compensate for the lack of PI3K activity in T cells (Okkenhaug et al., 2002; Garcon et al., 2008; Gogishvili et al., 2008). PDK1^−/−^ T cells showed reduced conversion to iTreg *in vitro* and *in vivo* (Park et al., 2010) while more iTregs developed upon stable transgenic expression of active Akt (Pierau et al., 2009). These latter studies suggest that PI3K pathway activation is required for iTreg differentiation, possibly at the level of initial activation, stabilization of Foxp3 expression, or survival of Foxp3^+^ cells. However, TGF-β-dependent conversion was reduced upon deletion of Pten, PHLPP or Foxo transcription factors, or by retroviral expression of Akt (Haxhinasto et al., 2008; Sauer et al., 2008; Patterson et al., 2011). Therefore, very high PI3K-Akt activity may be incompatible with iTreg conversion, presumably because it would eliminate Foxo from the nucleus.

Foxp3 expression also can be induced in naïve CD4^+^ T cells independently of TGF-β1 by removing cells from TCR stimuli 18 h after initial activation *in vitro* (Sauer et al., 2008). Interestingly, this effect could be enhanced by the addition of PI3K and mTOR inhibitors also added 18 h after activation (Sauer et al., 2008). TCR deprivation could not induce Foxp3 expression in Foxo1^−/−^Foxo3^−/−^ T cells, suggesting that the effect of the PI3K inhibitors depends on nuclear expression of Foxo (Ouyang et al., 2010).

## PI3K and mTOR inhibition have opposing effects on peripheral Treg homeostasis and expansion

Once Foxp3 is expressed, Tregs must process external cues in order to be maintained. One indicator of intact maintenance is the preservation of normal levels of Tregs under homeostatic conditions. There were 2-fold fewer peripheral Tregs in p110δ^D910A^ mice despite increased nTreg generation (Patton et al., 2006). This implies that PI3K signals are important for maintenance of Tregs. Consistent with this, Pten^−/−^ Treg show enhanced proliferation in response to IL-2 and mice in which Pten was deleted in Treg (as well as activated CD4^+^ T cells) have increased numbers of peripheral Treg (Walsh et al., 2006; Soond et al., 2012). Although Tregs with inactive p110δ proliferated normally in responses to IL-2 *in vitro* (Patton et al., 2006), it is possible that integrated signals from the TCR and IL-2R fail to support normal Treg numbers in p110δ^D910A^ mice *in vivo*. Mice with Foxo1 and Foxo3 deleted in T cells show a gradual recovery of Treg numbers with age, suggesting that Foxo may play a more important role in initial development of Tregs than in their maintenance in the periphery (Kerdiles et al., 2010).

Deprivation of mTOR signals by rapamycin or by deleting the gene encoding the mTOR catalytic subunit in T cells favors the expansion of Tregs (Battaglia et al., 2005; Delgoffe et al., 2009). Immunization with very low peptide concentrations promotes Treg differentiation and this is enhanced by rapamycin (Daniel et al., 2010). Whether mTOR inhibition actually enhances Treg expansion or gives Treg a selective growth advantage over other Th cell lineages remains a subject of debate. In a further twist, a recent study has also shown reduced expansion of Foxp3^−^ T cells after transfer of Foxp3^+^ T cells into lymphopenic hosts (Yurchenko et al., 2012). Whether this represented true reprogramming or these apparently converted Foxp3^−^ T cells were derived from contaminating Foxp3^−^ progenitors or partially differentiated Foxp3^+^ cells could not be established conclusively.

There are a number of potential mechanisms that render Treg insensitive, or even activated, by mTOR inhibition. Tregs express high levels of the serine-threonine kinase Pim2, which shares many targets in common with mTOR (Basu et al., 2008). Prolonged treatment with rapamycin can partially inhibit phosphorylation of Akt and hence enhance nuclear retention of Foxo (Sarbassov et al., 2006). Rapamycin can also mimic the effect of amino acid deprivation which favors Treg expansion (Cobbold et al., 2009). Furthermore, in contrast to Th, Tregs depend highly on lipid oxidation rather than glycolysis. By inhibiting mTOR-dependent glycolysis in favor of lipid oxidation, rapamycin may favor the expansion of Tregs (Michalek et al., 2011; Shi et al., 2011).

## Tregs have many mechanisms to suppress immune responses

Perhaps more important than the role in the development and maintenance is the question of whether PI3K signaling controls Treg-mediated suppression. Tregs provide a dominant mechanism of peripheral tolerance and hence moderate variations in their absolute numbers do not necessarily have significant impacts on their ability to prevent disease. Rather, it is the amount of Foxp3 expressed per Treg that is essential (Wan and Flavell, 2007).

Tregs employ a variety of mechanisms to suppress the immune system, and these differ whether the Tregs are suppressing immune responses elicited by self-antigens or commensal bacteria (Yamaguchi et al., 2011). Tregs can secrete suppressive cytokines such as IL-10, TGF-β, and IL-35 (Read et al., 2000; Collison et al., 2007; Rubtsov et al., 2008) which directly inhibit T cells and accessory leukocytes. IL-10 is critical in the gut, as mice with a Treg-specific deficiency in IL-10 develop inflammatory bowel disease, but not systemic autoimmunity (Rubtsov et al., 2008). Tregs constitutively express high levels of the IL-2R, and they can deprive conventional T cells of access to IL-2 (Pandiyan et al., 2007, 2011). CTLA-4 is also essential for Treg suppression, as Treg-specific CTLA-4 knockout mice succumb to a severe autoimmune syndrome (Wing et al., 2008). CTLA-4 blocks T cell activation by physically removing CD80 and CD86 from dendritic cells, thus depriving effector T cells of costimulation (Yokosuka et al., 2010; Qureshi et al., 2011). CTLA-4 binding can also instruct dendritic cells to release indoleamine 2,3-dioxygenase, which produces pro-apoptotic kyneurenines and deprives proliferating T cells of the tryptophan needed for growth (Grohmann et al., 2002). Tregs can transfer the inhibitory second messenger cAMP via gap junctions (Bopp et al., 2007), alter levels of extracellular nucleotides using CD38, CD39, and CD73 (Chen et al., 2006; Deaglio et al., 2007; Hubert et al., 2010), and kill activated leukocytes through the release of perforin or granzyme (Cao et al., 2007; Boissonnas et al., 2010). However, no single mechanism has been identified which either is unique to Tregs or which accounts for all aspects of Treg-mediated suppression (Yamaguchi et al., 2011; Josefowicz et al., 2012a).

## PI3K promotes Treg-mediated suppression

p110δ^D910A^ Tregs produce less IL-10 and show reduced suppression of CD4^+^ T cell proliferation *in vitro* (Patton et al., 2006, 2011). The reduced ability of p110δ-deficient Treg to supress was correlated with lower expression of CD38, a marker of a highly suppressive Treg population in the gut, suggesting that “effector” Tregs may not develop normally in p110δ^D910A^ mice (Cretney et al., 2011; Patton et al., 2011). Tregs from p110δ^D910A^ cannot block the development of experimental colitis induced by transfer of naïve T cells in Rag knockout mice (Patton et al., 2006). Consistent with this, p110δ^D910A^ mice spontaneously develop colitis of similar severity to that observed in IL-10-deficient mice (Okkenhaug et al., 2002; Uno et al., 2010). IL-10^−/−^p110δ^D910A^ double deficient mice develop a more severe form of colitis (Uno et al., 2010), suggesting that the disease caused by p110δ-deficiency is not only a consequence of impaired IL-10 production. PDK1^−/−^ Tregs had multiple functional defects that resulted in the failure to suppress γδ T cell-dependent colitis, again suggesting a key role for PI3K signaling in suppression of gut-associated inflammation (Park et al., 2010). Mice lacking p85 regulatory subunits in T cells develop Sjogrens syndrome, perhaps also as a consequence of impaired Treg function, although this mechanism was not examined directly (Oak et al., 2006). In the context of infection, p110δ^D910A^ mice showed a surprising increased ability to eliminate *Leishmania* parasites despite mounting a reduced Th1 response. This was explained, at least in part, by reduced Treg expansion and/or function in p110δ^D910A^ mice as the transfer of Treg into p110δ^D910A^ mice reversed their resistance to *Leishmania* (Liu et al., 2009). It should be noted that so far, no evidence of autoimmunity has been described in PI3K-deficient mice, suggesting that defective Treg function in these mice primarily affect responses to commensal or pathogenic organisms.

Both SHIP^−/−^ and Pten^−/−^ Treg suppressed normally *in vitro* and *in vivo* suggesting that moderately increased levels of PI3K activity is compatible with normal Treg function (Kashiwada et al., 2006; Walsh et al., 2006; Collazo et al., 2009; Locke et al., 2009; Patterson et al., 2011; Soond et al., 2012). However, Tregs overexpressing active Akt or in which the Akt-phosphatase PHLPP were deleted showed reduced capacity to suppress CD4^+^ T cell proliferation (Crellin et al., 2007; Patterson et al., 2011). Thus, some PI3K activity is required for optimal Treg-mediated suppression, but very high Akt activity can inhibit Treg suppression.

## Conclusions

Does the PI3K pathway promote or antagonize regulatory T cell development and function? The answer seems to be: both.

**Figure 1:**
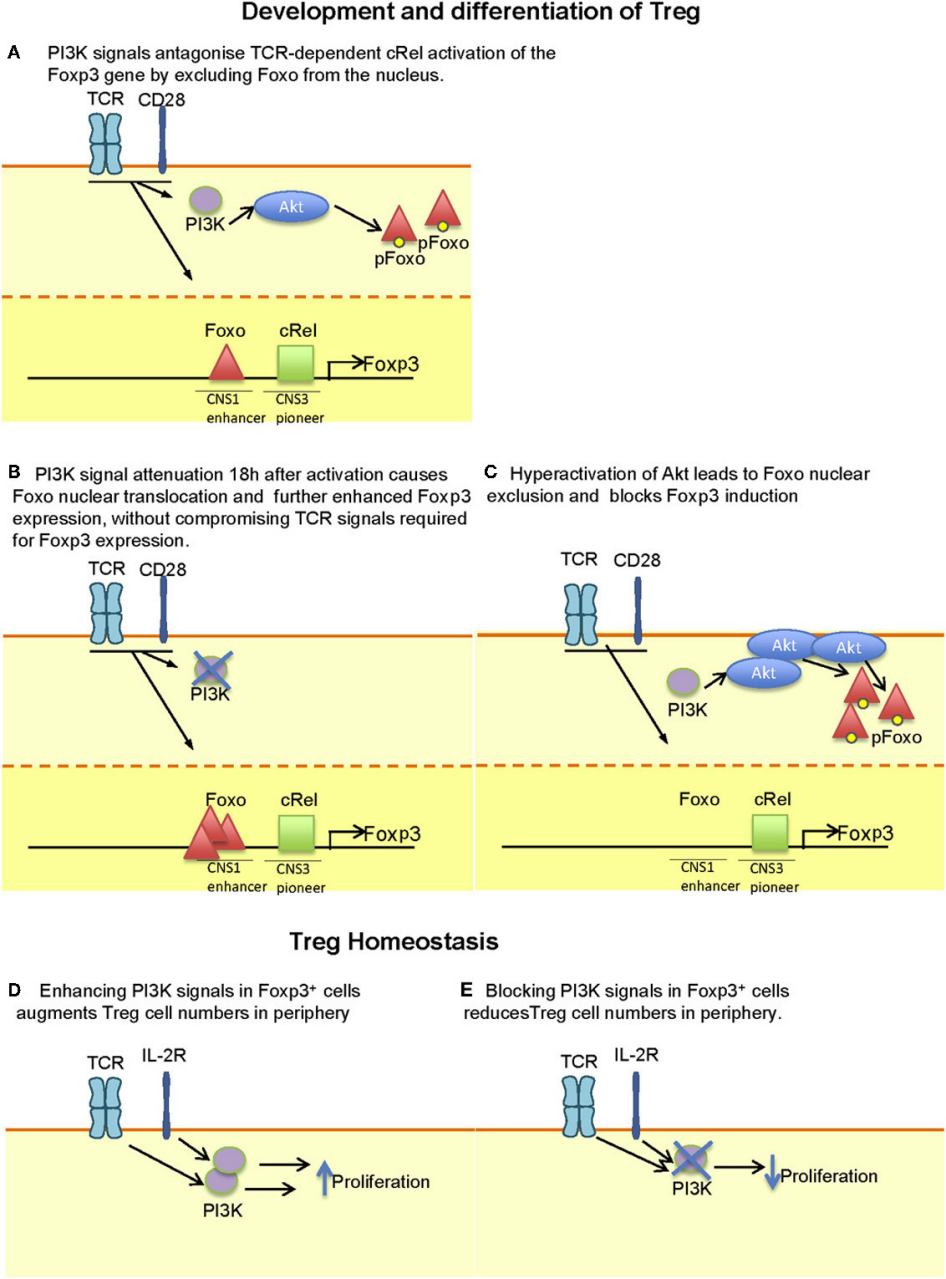
**Context-dependent effect of PI3K signaling on Treg development, differentiation and maintenance. (A)** During thymic development, attenuation of PI3K signaling may be required to enhance the translocation of Foxo proteins to the nucleus where they enhance the expression of Foxp3. **(B)** The capacity of PI3K signaling to antagonize is illustrated during the *in vitro* differentiation of Foxp3^−^ CD4^+^ T cells to Foxp3^+^ T cells where signal attenuation after 18 h facilitates Foxo nuclear translocation. It is worth noting that earlier inhibition may interfere with TCR-dependent signals that favor Foxp3 expression. **(C)** Hyperactivation of Akt (e.g. by expression of a membrane targeted Akt transgene) leads to Foxo nuclear exclusion and reduced Foxp3 expression. Whether such extent of Akt activation can be achieved by physiological receptor activation has yet to be determined. **(D)** Treg numbers in the periphery is controlled by both TCR and IL-2 signals. Inhibition of PI3K is likely to reduce the ability of these receptors to maintain Treg homeostasis, thus explaining the reduced number of Treg in p110δ-deficient mice. **(E)** Enhanced numbers of Treg are found upon deletion of Pten or Ship in T cells in which PI3K signaling is enhanced, probably reflecting enhanced IL-2-dependent homeostatic expansion.

The findings that the *Foxp3* gene contains Foxo-binding elements in its promoter region, along with the observation that PI3K inhibitors added 18 h after activation could be used to induce Treg differentiation raised the possibility that PI3K inhibitors could be used to enhance Treg induction *in vivo* (Bruno and Merkenschlager, 2008; Ohkura et al., 2011). However, inhibition of PI3K signaling in mice does not lead to increased numbers of peripheral Treg, even when deleted after activation using OX40-Cre (Rolf et al., 2010). Moreover, the evidence for enhanced Treg proportions or numbers in patients taking rapamycin or its analogs is lacking, despite the demonstrated property of rapamycin to favor the expansion of established Treg *in vitro* and *in vivo* (Zuber et al., 2011). Figure 1 illustrates how PI3K signaling can both antagonize and augment Treg numbers, depending on the stage of differentiation, timing, and extent of activation/inactivation.

The development of colitis and Sjogren's syndrome in mice with chronic inhibition of the PI3K pathway suggests that, on balance, inhibition of PI3K is more likely to inhibit Treg function than enhance it. It is therefore unlikely that patients who are administered p110δ-selective inhibitors would have enhanced Treg function. Whether impairment in Treg function or homeostasis leading to a clinical manifestation will be a significant detrimental side effect in patients who are administered PI3K inhibitors is not yet clear; however, serious side effects have not been reported in initial clinical trials so far (Furman et al., 2010; Fruman and Rommel, 2011). Indeed, protective effects of p110δ inhibition in mouse models of asthma, multiple sclerosis, arthritis, and lupus suggest that blockade of effector T cells dominates (Nashed et al., 2007; Durand et al., 2009; Haylock-Jacobs et al., 2011). A number of pharmaceutical companies are also developing p110δ or p110γ inhibitors to treat leukemia or autoimmune diseases (Fruman and Rommel, 2011; Norman, 2011; So and Fruman, 2012). The first publically available results from clinical trials using p110δ inhibitors suggest a remarkable response rate in patients with chronic lymphocytic leukemia, nearly all of whom showed reduced lymph node size after treatments in phase I trials (Furman et al., 2010). Could inhibition of Treg by p110δ inhibitors be exploited therapeutically? One positive outcome of reduced Treg function was shown in p110δ^D910A^ mice which were resistant to infection with *Leishmania* (Liu et al., 2009). A recent study suggested a beneficial effect of PI3K inhibitors as adjuvants to cancer vaccines (Marshall et al., 2012). We are currently exploring whether p110δ-inhibition of Treg function could be used to enhance anti-tumor responses. In summary, inhibiting PI3K can facilitate the differentiation of Treg *in vitro*, but *in vivo*, the net results of PI3K inhibition is fewer Tregs with reduced, but not abolished, suppressive capacity.

### Conflict of interest statement

Klaus Okkenhaug is paid consultant for GlaxoSmithKline. Other authors declare that the research was conducted in the absence of any commercial or financial relationships that could be construed as a potential conflict of interest.
